# *N*,*N*,*N*′-Tris[(1*H*-indol-3-yl)meth­yl]ethane-1,2-di­amine

**DOI:** 10.1107/S1600536813028225

**Published:** 2013-10-19

**Authors:** Yan-Jun Li, De-Ju Wang

**Affiliations:** a Wuhan University of Science and Technology, Hubei, Wuhan 430081, People’s Republic of China

## Abstract

In the title mol­ecule, C_29_H_29_N_5_, the indole ring systems are essentially planar, with maximum deviations of 0.020 (2), 0.023 (2) and 0.016 (2) Å. The dihedral angles formed between the mean planes of the three indole ring systems are 38.08 (7), 89.64 (8) and 58.28 (8)°. In the crystal, mol­ecules are connected by N—H⋯N hydrogen bonds, forming inversion dimers. An intra­molecular N—H⋯N hydrogen bond is also observed.

## Related literature

For applications of indole compounds and for related structures, see: Shimazaki *et al.*(2009 ▶); Takani *et al.* (2006 ▶); Munjal *et al.* (2010 ▶); Zhu *et al.* (2012 ▶).
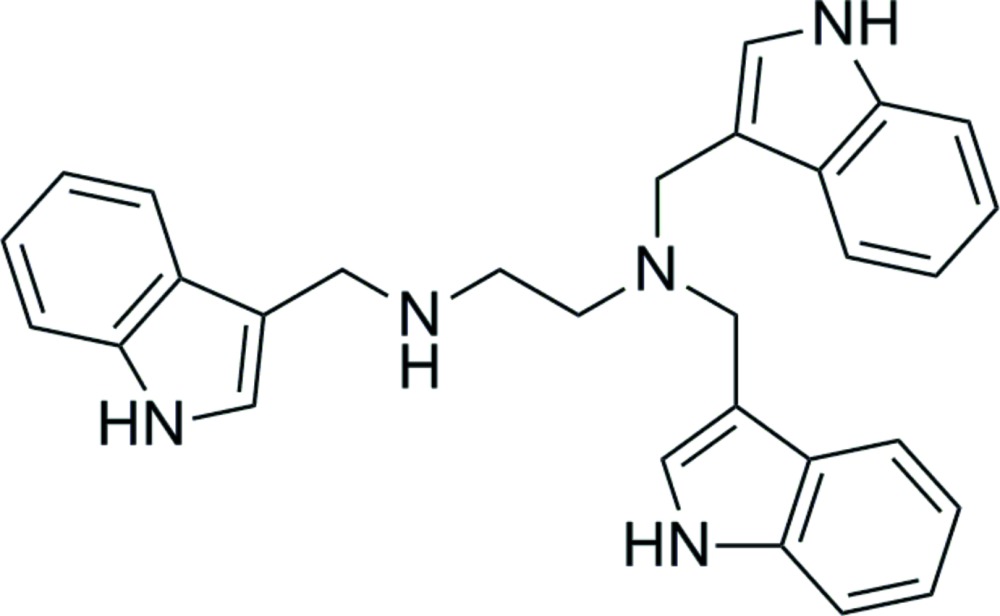


## Experimental

### 

#### Crystal data

C_29_H_29_N_5_
*M* *_r_* = 447.57
Monoclinic, 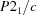

*a* = 13.769 (3) Å
*b* = 10.832 (2) Å
*c* = 16.910 (3) Å
β = 106.512 (3)°
*V* = 2418.2 (8) Å^3^
*Z* = 4
Mo *K*α radiation
μ = 0.07 mm^−1^
*T* = 298 K
0.16 × 0.15 × 0.10 mm

#### Data collection

Bruker SMART CCD diffractometer
Absorption correction: multi-scan (*SADABS*; Bruker, 2001 ▶) *T*_min_ = 0.988, *T*_max_ = 0.993
12582 measured reflections
4486 independent reflections
2785 reflections with *I* > 2σ(*I*)
*R*_int_ = 0.032

#### Refinement

*R*[*F* ^2^ > 2σ(*F* ^2^)] = 0.044
*wR*(*F* ^2^) = 0.114
*S* = 0.99
4486 reflections
319 parameters
4 restraints
H atoms treated by a mixture of independent and constrained refinement
Δρ_max_ = 0.12 e Å^−3^
Δρ_min_ = −0.17 e Å^−3^

### 

Data collection: *SMART* (Bruker, 2001 ▶); cell refinement: *SAINT* (Bruker, 2001 ▶); data reduction: *SAINT*; program(s) used to solve structure: *SHELXS97* (Sheldrick, 2008 ▶); program(s) used to refine structure: *SHELXL97* (Sheldrick, 2008 ▶); molecular graphics: *SHELXTL* (Sheldrick, 2008 ▶); software used to prepare material for publication: *SHELXTL*.

## Supplementary Material

Crystal structure: contains datablock(s) I, New_Global_Publ_Block. DOI: 10.1107/S1600536813028225/lh5652sup1.cif

Structure factors: contains datablock(s) I. DOI: 10.1107/S1600536813028225/lh5652Isup2.hkl

Supplementary material file. DOI: 10.1107/S1600536813028225/lh5652Isup3.cml

Additional supplementary materials:  crystallographic information; 3D view; checkCIF report

## Figures and Tables

**Table 1:** Hydrogen-bond geometry (Å, °)

*D*—H⋯*A*	*D*—H	H⋯*A*	*D*⋯*A*	*D*—H⋯*A*
N4—H4⋯N2^i^	0.92 (2)	2.03 (2)	2.953 (3)	176 (2)
N2—H2⋯N1	0.83 (2)	2.45 (2)	2.889 (2)	114 (2)

Symmetry code: (i) 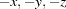.

**Fig. 1.**
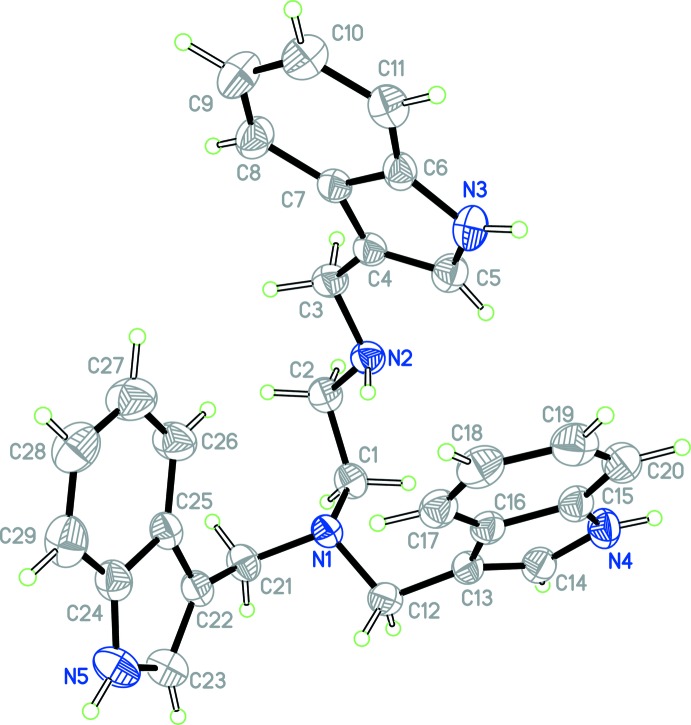
The molecular structure of (I), with 30% probability displacement ellipsoids for non-H atoms.

## supplementary crystallographic information

### 1. Comment

The indole group is a benzo-fused, electron rich aromatic compound widely distributed in natural products, which show important biological activities (Shimazaki *et al.*, 2009). Design and synthesis of new transition metal complexes including indole groups are very interesting and important for bioinorganic and bioorganic chemistry. The indole ring system has been shown to have versatile metal binding abilities involving a metal ion with both nitrogen and carbon atoms (Takani *et al.*2006; Munjal *et al.*2010; Zhu *et al.*2012). During the condensation reaction of ethylenediamine and 1*H*-indole-3-carbaldehyde, the title compound was obtained as a side-product. Herein we report the crystal structure of the title compound (I).

The molecular structure of (I) is shown in Fig. 1. The indole ring systems are essentially planar with maximum deviations in each ring of 0.020 (2), 0.023 (2) and 0.016 (2)Å for N3, C19 and C22, respectively. The dihedral angles formed between the three indole ring systems are 38.08 (7)° for C4-C11/N3 and C13-C20/N4, 89.64 (8)° for C4-C11/N3 and C22-C29/N5, and 58.28 (8)° for C13-C20/N4 and C22-C29/N5. In the crystal, molecules are connected by N—H···N hydrogen bonds, forming inversion dimers. An intramolecular N—H···N hydrogen bond is also observed (Table 1). .

### 2. Experimental

Ethylenediamine (0.6 g, 10 mmol) and 1*H*-indole-3-carbaldehyde (2.9 g, 20 mmol) were dissolved in methanol (50 ml), sodium cyanoborohydride (2.5 g,40 mmol) was then added and the solution stirring at room temperature for 36 h. The mixture was then poured onto water and extracted with dichloromethane twice. The organic layer was washed with brine, dried over with MgSO_4_, filtered, and the solvent was removed under reduced pressure. Flash chromatography on a silica column with methanol/dichloromthane (1:3,%*v*/*v*) and then recrystallization from methanol solution gave colourless crystals of the title compound (1.35 g,29% yield based on the ethylenediamine).

### 3. Refinement

All carbon-bound H atoms were constrained to their expected geometries [C—H 0.93 and 0.97 Å] and refined with *U*_iso_(H) = 1.2*U*_eq_(C). H atoms bonded to N atoms were refined with the constraint N—H = 0.82 (2)Å and *U*_iso_(H) = 1.2*U*_eq_(N).

### Crystal data

**Table:**

C_29_H_29_N_5_	*F*(000) = 952
*M**_r_* = 447.57	*D*_x_ = 1.229 Mg m^−^^3^
Monoclinic, *P*2_1_/*c*	Mo *K*α radiation, λ = 0.71073 Å
Hall symbol: -P 2ybc	Cell parameters from 2164 reflections
*a* = 13.769 (3) Å	θ = 2.4–20.2°
*b* = 10.832 (2) Å	µ = 0.07 mm^−^^1^
*c* = 16.910 (3) Å	*T* = 298 K
β = 106.512 (3)°	Block, colourless
*V* = 2418.2 (8) Å^3^	0.16 × 0.15 × 0.10 mm
*Z* = 4	

### Data collection

**Table:**

Bruker SMART CCD diffractometer	4486 independent reflections
Radiation source: fine-focus sealed tube	2785 reflections with *I* > 2σ(*I*)
Graphite monochromator	*R*_int_ = 0.032
φ and ω scans	θ_max_ = 25.5°, θ_min_ = 2.3°
Absorption correction: multi-scan (*SADABS*; Bruker, 2001)	*h* = −15→16
*T*_min_ = 0.988, *T*_max_ = 0.993	*k* = −13→8
12582 measured reflections	*l* = −19→20

### Refinement

**Table:**

Refinement on *F*^2^	Primary atom site location: structure-invariant direct methods
Least-squares matrix: full	Secondary atom site location: difference Fourier map
*R*[*F*^2^ > 2σ(*F*^2^)] = 0.044	Hydrogen site location: inferred from neighbouring sites
*wR*(*F*^2^) = 0.114	H atoms treated by a mixture of independent and constrained refinement
*S* = 0.99	*w* = 1/[σ^2^(*F*_o_^2^) + (0.056*P*)^2^] where *P* = (*F*_o_^2^ + 2*F*_c_^2^)/3
4486 reflections	(Δ/σ)_max_ < 0.001
319 parameters	Δρ_max_ = 0.12 e Å^−^^3^
4 restraints	Δρ_min_ = −0.17 e Å^−^^3^

### Special details

**Table:**

Geometry. All e.s.d.'s (except the e.s.d. in the dihedral angle between two l.s. planes) are estimated using the full covariance matrix. The cell e.s.d.'s are taken into account individually in the estimation of e.s.d.'s in distances, angles and torsion angles; correlations between e.s.d.'s in cell parameters are only used when they are defined by crystal symmetry. An approximate (isotropic) treatment of cell e.s.d.'s is used for estimating e.s.d.'s involving l.s. planes.
Refinement. Refinement of *F*^2^ against ALL reflections. The weighted *R*-factor *wR* and goodness of fit *S* are based on *F*^2^, conventional *R*-factors *R* are based on *F*, with *F* set to zero for negative *F*^2^. The threshold expression of *F*^2^ > σ(*F*^2^) is used only for calculating *R*-factors(gt) *etc*. and is not relevant to the choice of reflections for refinement. *R*-factors based on *F*^2^ are statistically about twice as large as those based on *F*, and *R*- factors based on ALL data will be even larger.

### Fractional atomic coordinates and isotropic or equivalent isotropic displacement parameters (Å^2^)

**Table:**

	*x*	*y*	*z*	*U*_iso_*/*U*_eq_	
C1	0.21660 (15)	0.20856 (19)	−0.00132 (12)	0.0548 (5)	
H1A	0.2423	0.2468	−0.0432	0.066*	
H1B	0.1526	0.1692	−0.0287	0.066*	
C2	0.29084 (15)	0.11279 (19)	0.04444 (12)	0.0560 (5)	
H2A	0.3045	0.0538	0.0058	0.067*	
H2B	0.3542	0.1521	0.0736	0.067*	
C3	0.32167 (15)	−0.0165 (2)	0.16844 (13)	0.0572 (6)	
H3A	0.3782	0.0377	0.1932	0.069*	
H3B	0.3475	−0.0868	0.1451	0.069*	
C4	0.27550 (14)	−0.06000 (18)	0.23321 (12)	0.0490 (5)	
C5	0.17503 (15)	−0.07432 (19)	0.22497 (13)	0.0569 (6)	
H5	0.1234	−0.0572	0.1773	0.068*	
C6	0.25247 (14)	−0.13274 (18)	0.35366 (12)	0.0496 (5)	
C7	0.32704 (14)	−0.09563 (17)	0.31615 (12)	0.0488 (5)	
C8	0.42825 (16)	−0.0998 (2)	0.36324 (14)	0.0646 (6)	
H8	0.4796	−0.0758	0.3408	0.078*	
C9	0.45039 (17)	−0.1402 (2)	0.44351 (15)	0.0749 (7)	
H9	0.5176	−0.1418	0.4755	0.090*	
C10	0.37555 (17)	−0.1784 (2)	0.47800 (14)	0.0685 (6)	
H10	0.3934	−0.2066	0.5322	0.082*	
C11	0.27581 (16)	−0.1753 (2)	0.43353 (13)	0.0599 (6)	
H11	0.2253	−0.2012	0.4565	0.072*	
C12	0.09756 (14)	0.35402 (19)	0.03092 (13)	0.0559 (6)	
H12A	0.0811	0.3816	−0.0259	0.067*	
H12B	0.0945	0.4251	0.0651	0.067*	
C13	0.02160 (14)	0.26081 (19)	0.03937 (12)	0.0507 (5)	
C14	−0.04813 (15)	0.2018 (2)	−0.02227 (13)	0.0579 (6)	
H14	−0.0576	0.2164	−0.0782	0.069*	
C15	−0.06722 (15)	0.12209 (19)	0.09287 (13)	0.0537 (5)	
C16	0.01110 (14)	0.21007 (18)	0.11443 (12)	0.0499 (5)	
C17	0.06024 (15)	0.2313 (2)	0.19794 (13)	0.0589 (6)	
H17	0.1134	0.2874	0.2135	0.071*	
C18	0.02882 (18)	0.1681 (2)	0.25622 (14)	0.0696 (7)	
H18	0.0610	0.1816	0.3118	0.084*	
C19	−0.05100 (19)	0.0836 (2)	0.23338 (16)	0.0726 (7)	
H19	−0.0718	0.0433	0.2743	0.087*	
C20	−0.09945 (17)	0.0584 (2)	0.15233 (16)	0.0670 (6)	
H20	−0.1518	0.0011	0.1376	0.080*	
C21	0.27573 (16)	0.40222 (19)	0.06586 (12)	0.0564 (6)	
H21A	0.2544	0.4589	0.0198	0.068*	
H21B	0.3398	0.3664	0.0645	0.068*	
C22	0.29158 (14)	0.47297 (19)	0.14409 (12)	0.0517 (5)	
C23	0.27479 (18)	0.5946 (2)	0.15314 (15)	0.0667 (6)	
H23	0.2451	0.6483	0.1102	0.080*	
C24	0.34721 (16)	0.5248 (2)	0.28034 (14)	0.0588 (6)	
C25	0.33709 (15)	0.42618 (19)	0.22502 (12)	0.0509 (5)	
C26	0.37186 (17)	0.3112 (2)	0.25615 (14)	0.0704 (6)	
H26	0.3658	0.2439	0.2210	0.084*	
C27	0.4153 (2)	0.2975 (3)	0.33937 (16)	0.0852 (8)	
H27	0.4383	0.2201	0.3604	0.102*	
C28	0.42564 (19)	0.3970 (3)	0.39261 (15)	0.0812 (8)	
H28	0.4563	0.3854	0.4487	0.097*	
C29	0.39172 (18)	0.5112 (3)	0.36443 (15)	0.0750 (7)	
H29	0.3981	0.5778	0.4003	0.090*	
N1	0.20034 (11)	0.30344 (14)	0.05540 (9)	0.0476 (4)	
N2	0.24795 (13)	0.04946 (16)	0.10288 (11)	0.0525 (5)	
H2	0.2211 (15)	0.1029 (17)	0.1248 (12)	0.063*	
N3	0.16084 (13)	−0.11738 (18)	0.29652 (11)	0.0610 (5)	
H3	0.1064 (14)	−0.135 (2)	0.3083 (13)	0.073*	
N4	−0.10242 (13)	0.11849 (18)	0.00839 (12)	0.0606 (5)	
H4	−0.1491 (16)	0.066 (2)	−0.0244 (12)	0.073*	
N5	0.30761 (17)	0.62689 (19)	0.23432 (14)	0.0758 (6)	
H5A	0.3038 (18)	0.6959 (18)	0.2547 (15)	0.091*	

### Atomic displacement parameters (Å^2^)

**Table:**

	*U*^11^	*U*^22^	*U*^33^	*U*^12^	*U*^13^	*U*^23^
C1	0.0607 (13)	0.0539 (13)	0.0514 (12)	−0.0051 (11)	0.0184 (10)	−0.0059 (10)
C2	0.0567 (12)	0.0555 (13)	0.0594 (13)	−0.0005 (11)	0.0226 (11)	−0.0058 (11)
C3	0.0540 (12)	0.0495 (13)	0.0697 (14)	0.0061 (10)	0.0202 (11)	0.0013 (11)
C4	0.0474 (12)	0.0402 (11)	0.0601 (13)	0.0007 (9)	0.0166 (10)	−0.0032 (10)
C5	0.0516 (13)	0.0594 (14)	0.0579 (14)	0.0006 (11)	0.0124 (10)	−0.0064 (11)
C6	0.0455 (11)	0.0448 (12)	0.0587 (13)	−0.0032 (10)	0.0153 (10)	−0.0102 (10)
C7	0.0474 (11)	0.0388 (11)	0.0602 (13)	0.0012 (9)	0.0152 (10)	−0.0040 (10)
C8	0.0466 (12)	0.0747 (16)	0.0742 (16)	0.0027 (11)	0.0198 (11)	0.0008 (13)
C9	0.0512 (13)	0.100 (2)	0.0687 (16)	0.0086 (13)	0.0087 (11)	0.0051 (14)
C10	0.0651 (15)	0.0786 (18)	0.0621 (14)	0.0072 (13)	0.0188 (12)	0.0015 (12)
C11	0.0617 (13)	0.0584 (14)	0.0629 (15)	−0.0043 (12)	0.0230 (11)	−0.0054 (11)
C12	0.0572 (13)	0.0482 (13)	0.0577 (13)	0.0010 (11)	0.0088 (10)	−0.0006 (10)
C13	0.0462 (11)	0.0469 (12)	0.0552 (13)	0.0008 (10)	0.0086 (9)	−0.0065 (10)
C14	0.0528 (12)	0.0604 (14)	0.0586 (13)	0.0011 (11)	0.0131 (10)	−0.0038 (11)
C15	0.0482 (12)	0.0524 (13)	0.0605 (14)	0.0021 (10)	0.0156 (10)	−0.0074 (11)
C16	0.0452 (11)	0.0468 (12)	0.0570 (13)	0.0059 (10)	0.0135 (9)	−0.0068 (10)
C17	0.0527 (12)	0.0605 (14)	0.0607 (14)	0.0054 (11)	0.0116 (10)	−0.0093 (11)
C18	0.0697 (15)	0.0787 (17)	0.0596 (15)	0.0148 (14)	0.0169 (12)	−0.0015 (13)
C19	0.0759 (16)	0.0734 (17)	0.0773 (18)	0.0112 (14)	0.0361 (14)	0.0081 (14)
C20	0.0600 (14)	0.0608 (15)	0.0851 (18)	0.0016 (12)	0.0287 (13)	−0.0036 (13)
C21	0.0630 (13)	0.0547 (13)	0.0535 (13)	−0.0103 (11)	0.0196 (10)	0.0005 (10)
C22	0.0518 (12)	0.0465 (13)	0.0583 (13)	−0.0074 (10)	0.0182 (10)	−0.0045 (10)
C23	0.0782 (16)	0.0544 (15)	0.0695 (16)	0.0008 (12)	0.0243 (12)	−0.0005 (12)
C24	0.0567 (13)	0.0581 (15)	0.0648 (15)	−0.0044 (12)	0.0224 (11)	−0.0143 (12)
C25	0.0513 (12)	0.0481 (13)	0.0532 (13)	−0.0072 (10)	0.0146 (10)	−0.0078 (10)
C26	0.0890 (17)	0.0563 (15)	0.0599 (15)	0.0042 (13)	0.0114 (12)	−0.0058 (12)
C27	0.102 (2)	0.0800 (19)	0.0637 (17)	0.0137 (16)	0.0079 (14)	0.0055 (14)
C28	0.0788 (17)	0.105 (2)	0.0564 (15)	0.0071 (16)	0.0134 (13)	−0.0044 (16)
C29	0.0732 (16)	0.092 (2)	0.0612 (16)	−0.0056 (15)	0.0221 (12)	−0.0276 (14)
N1	0.0498 (10)	0.0429 (10)	0.0498 (10)	−0.0044 (8)	0.0139 (8)	−0.0041 (8)
N2	0.0521 (10)	0.0478 (11)	0.0598 (11)	0.0021 (9)	0.0193 (8)	−0.0007 (9)
N3	0.0455 (10)	0.0755 (14)	0.0633 (12)	−0.0074 (10)	0.0176 (9)	−0.0044 (10)
N4	0.0521 (10)	0.0605 (12)	0.0669 (13)	−0.0107 (9)	0.0129 (9)	−0.0135 (10)
N5	0.0988 (15)	0.0515 (13)	0.0811 (16)	0.0023 (12)	0.0318 (12)	−0.0176 (12)

### Geometric parameters (Å, º)

**Table:**

C1—N1	1.466 (2)	C15—N4	1.373 (3)
C1—C2	1.507 (3)	C15—C20	1.392 (3)
C1—H1A	0.9700	C15—C16	1.407 (3)
C1—H1B	0.9700	C16—C17	1.401 (3)
C2—N2	1.459 (2)	C17—C18	1.367 (3)
C2—H2A	0.9700	C17—H17	0.9300
C2—H2B	0.9700	C18—C19	1.398 (3)
C3—N2	1.459 (3)	C18—H18	0.9300
C3—C4	1.491 (3)	C19—C20	1.370 (3)
C3—H3A	0.9700	C19—H19	0.9300
C3—H3B	0.9700	C20—H20	0.9300
C4—C5	1.359 (3)	C21—N1	1.466 (2)
C4—C7	1.433 (3)	C21—C22	1.490 (3)
C5—N3	1.362 (3)	C21—H21A	0.9700
C5—H5	0.9300	C21—H21B	0.9700
C6—N3	1.364 (3)	C22—C23	1.354 (3)
C6—C11	1.376 (3)	C22—C25	1.427 (3)
C6—C7	1.410 (3)	C23—N5	1.363 (3)
C7—C8	1.396 (3)	C23—H23	0.9300
C8—C9	1.375 (3)	C24—N5	1.373 (3)
C8—H8	0.9300	C24—C29	1.387 (3)
C9—C10	1.384 (3)	C24—C25	1.400 (3)
C9—H9	0.9300	C25—C26	1.383 (3)
C10—C11	1.366 (3)	C26—C27	1.372 (3)
C10—H10	0.9300	C26—H26	0.9300
C11—H11	0.9300	C27—C28	1.385 (3)
C12—N1	1.463 (2)	C27—H27	0.9300
C12—C13	1.490 (3)	C28—C29	1.360 (3)
C12—H12A	0.9700	C28—H28	0.9300
C12—H12B	0.9700	C29—H29	0.9300
C13—C14	1.359 (3)	N2—H2	0.829 (16)
C13—C16	1.428 (3)	N3—H3	0.849 (16)
C14—N4	1.365 (3)	N4—H4	0.92 (2)
C14—H14	0.9300	N5—H5A	0.832 (17)
			
N1—C1—C2	110.75 (16)	C15—C16—C13	107.10 (17)
N1—C1—H1A	109.5	C18—C17—C16	118.9 (2)
C2—C1—H1A	109.5	C18—C17—H17	120.5
N1—C1—H1B	109.5	C16—C17—H17	120.5
C2—C1—H1B	109.5	C17—C18—C19	120.9 (2)
H1A—C1—H1B	108.1	C17—C18—H18	119.5
N2—C2—C1	108.99 (16)	C19—C18—H18	119.5
N2—C2—H2A	109.9	C20—C19—C18	121.9 (2)
C1—C2—H2A	109.9	C20—C19—H19	119.1
N2—C2—H2B	109.9	C18—C19—H19	119.1
C1—C2—H2B	109.9	C19—C20—C15	117.3 (2)
H2A—C2—H2B	108.3	C19—C20—H20	121.3
N2—C3—C4	111.29 (16)	C15—C20—H20	121.3
N2—C3—H3A	109.4	N1—C21—C22	113.73 (16)
C4—C3—H3A	109.4	N1—C21—H21A	108.8
N2—C3—H3B	109.4	C22—C21—H21A	108.8
C4—C3—H3B	109.4	N1—C21—H21B	108.8
H3A—C3—H3B	108.0	C22—C21—H21B	108.8
C5—C4—C7	106.02 (18)	H21A—C21—H21B	107.7
C5—C4—C3	126.51 (19)	C23—C22—C25	106.25 (18)
C7—C4—C3	127.47 (17)	C23—C22—C21	127.9 (2)
C4—C5—N3	110.30 (19)	C25—C22—C21	125.62 (18)
C4—C5—H5	124.8	C22—C23—N5	110.2 (2)
N3—C5—H5	124.8	C22—C23—H23	124.9
N3—C6—C11	130.32 (19)	N5—C23—H23	124.9
N3—C6—C7	106.97 (18)	N5—C24—C29	130.9 (2)
C11—C6—C7	122.70 (18)	N5—C24—C25	106.7 (2)
C8—C7—C6	118.10 (19)	C29—C24—C25	122.4 (2)
C8—C7—C4	134.7 (2)	C26—C25—C24	118.3 (2)
C6—C7—C4	107.20 (17)	C26—C25—C22	134.03 (19)
C9—C8—C7	118.6 (2)	C24—C25—C22	107.65 (19)
C9—C8—H8	120.7	C27—C26—C25	119.4 (2)
C7—C8—H8	120.7	C27—C26—H26	120.3
C8—C9—C10	121.9 (2)	C25—C26—H26	120.3
C8—C9—H9	119.1	C26—C27—C28	121.1 (2)
C10—C9—H9	119.1	C26—C27—H27	119.4
C11—C10—C9	120.9 (2)	C28—C27—H27	119.4
C11—C10—H10	119.6	C29—C28—C27	121.2 (2)
C9—C10—H10	119.6	C29—C28—H28	119.4
C10—C11—C6	117.8 (2)	C27—C28—H28	119.4
C10—C11—H11	121.1	C28—C29—C24	117.6 (2)
C6—C11—H11	121.1	C28—C29—H29	121.2
N1—C12—C13	111.60 (16)	C24—C29—H29	121.2
N1—C12—H12A	109.3	C12—N1—C1	113.12 (15)
C13—C12—H12A	109.3	C12—N1—C21	110.83 (16)
N1—C12—H12B	109.3	C1—N1—C21	110.86 (15)
C13—C12—H12B	109.3	C2—N2—C3	114.63 (16)
H12A—C12—H12B	108.0	C2—N2—H2	107.1 (14)
C14—C13—C16	105.83 (18)	C3—N2—H2	107.7 (15)
C14—C13—C12	127.4 (2)	C5—N3—C6	109.50 (17)
C16—C13—C12	126.69 (17)	C5—N3—H3	130.0 (15)
C13—C14—N4	111.3 (2)	C6—N3—H3	120.5 (15)
C13—C14—H14	124.3	C14—N4—C15	107.88 (18)
N4—C14—H14	124.3	C14—N4—H4	123.2 (13)
N4—C15—C20	130.3 (2)	C15—N4—H4	128.7 (13)
N4—C15—C16	107.87 (19)	C23—N5—C24	109.17 (19)
C20—C15—C16	121.8 (2)	C23—N5—H5A	127.4 (18)
C17—C16—C15	119.16 (19)	C24—N5—H5A	123.4 (18)
C17—C16—C13	133.73 (19)		
			
N1—C1—C2—N2	63.4 (2)	C16—C15—C20—C19	−0.8 (3)
N2—C3—C4—C5	19.5 (3)	N1—C21—C22—C23	−118.0 (2)
N2—C3—C4—C7	−160.79 (18)	N1—C21—C22—C25	68.3 (3)
C7—C4—C5—N3	−0.6 (2)	C25—C22—C23—N5	0.5 (3)
C3—C4—C5—N3	179.15 (19)	C21—C22—C23—N5	−174.18 (19)
N3—C6—C7—C8	178.42 (18)	N5—C24—C25—C26	179.86 (19)
C11—C6—C7—C8	−1.6 (3)	C29—C24—C25—C26	0.8 (3)
N3—C6—C7—C4	−1.3 (2)	N5—C24—C25—C22	0.9 (2)
C11—C6—C7—C4	178.67 (18)	C29—C24—C25—C22	−178.12 (19)
C5—C4—C7—C8	−178.5 (2)	C23—C22—C25—C26	−179.6 (2)
C3—C4—C7—C8	1.8 (4)	C21—C22—C25—C26	−4.7 (4)
C5—C4—C7—C6	1.2 (2)	C23—C22—C25—C24	−0.9 (2)
C3—C4—C7—C6	−178.60 (19)	C21—C22—C25—C24	173.95 (18)
C6—C7—C8—C9	0.3 (3)	C24—C25—C26—C27	−0.4 (3)
C4—C7—C8—C9	179.8 (2)	C22—C25—C26—C27	178.2 (2)
C7—C8—C9—C10	1.1 (4)	C25—C26—C27—C28	−0.5 (4)
C8—C9—C10—C11	−1.2 (4)	C26—C27—C28—C29	1.0 (4)
C9—C10—C11—C6	−0.2 (4)	C27—C28—C29—C24	−0.5 (4)
N3—C6—C11—C10	−178.5 (2)	N5—C24—C29—C28	−179.1 (2)
C7—C6—C11—C10	1.6 (3)	C25—C24—C29—C28	−0.3 (3)
N1—C12—C13—C14	−108.4 (2)	C13—C12—N1—C1	68.4 (2)
N1—C12—C13—C16	67.8 (2)	C13—C12—N1—C21	−166.40 (16)
C16—C13—C14—N4	0.6 (2)	C2—C1—N1—C12	−147.38 (16)
C12—C13—C14—N4	177.40 (18)	C2—C1—N1—C21	87.41 (19)
N4—C15—C16—C17	−179.86 (17)	C22—C21—N1—C12	76.6 (2)
C20—C15—C16—C17	2.1 (3)	C22—C21—N1—C1	−156.94 (17)
N4—C15—C16—C13	0.5 (2)	C1—C2—N2—C3	−161.54 (17)
C20—C15—C16—C13	−177.50 (18)	C4—C3—N2—C2	170.19 (16)
C14—C13—C16—C17	179.8 (2)	C4—C5—N3—C6	−0.2 (2)
C12—C13—C16—C17	2.9 (3)	C11—C6—N3—C5	−179.0 (2)
C14—C13—C16—C15	−0.6 (2)	C7—C6—N3—C5	0.9 (2)
C12—C13—C16—C15	−177.50 (18)	C13—C14—N4—C15	−0.3 (2)
C15—C16—C17—C18	−1.7 (3)	C20—C15—N4—C14	177.6 (2)
C13—C16—C17—C18	177.8 (2)	C16—C15—N4—C14	−0.2 (2)
C16—C17—C18—C19	0.0 (3)	C22—C23—N5—C24	0.1 (3)
C17—C18—C19—C20	1.4 (3)	C29—C24—N5—C23	178.3 (2)
C18—C19—C20—C15	−1.0 (3)	C25—C24—N5—C23	−0.6 (3)
N4—C15—C20—C19	−178.2 (2)		

### Hydrogen-bond geometry (Å, º)

**Table:**

*D*—H···*A*	*D*—H	H···*A*	*D*···*A*	*D*—H···*A*
N4—H4···N2^i^	0.92 (2)	2.03 (2)	2.953 (3)	176 (2)
N2—H2···N1	0.83 (2)	2.45 (2)	2.889 (2)	114 (2)

Symmetry code: (i) −*x*, −*y*, −*z*.
